# Synthesis, Characterization, Crystal Structure and Antimicrobial Activity of Copper(II) Complexes with the Schiff Base Derived from 2-Hydroxy-4-Methoxybenzaldehyde

**DOI:** 10.3390/molecules20045771

**Published:** 2015-04-02

**Authors:** Elena Pahonțu, Diana-Carolina Ilieș, Sergiu Shova, Codruța Paraschivescu, Mihaela Badea, Aurelian Gulea, Tudor Roșu

**Affiliations:** 1 Inorganic Chemistry Department, Faculty of Pharmacy, University of Medicine and Pharmacy “Carol Davila”, 6 Traian Vuia Street, 020956 Bucharest, Romania; E-Mail: elenaandmihaela@yahoo.com; 2 Inorganic Chemistry Department, Faculty of Chemistry, University of Bucharest, 23 Dumbrava Rosie Street, 020462 Bucharest, Romania; E-Mails: e_m_badea@yahoo.com (M.B.); t_rosu0101@yahoo.com (T.R.); 3 Organic Chemistry Department, Faculty of Pharmacy, University of Medicine and Pharmacy “Carol Davila”, 6 Traian Vuia Street, 020956 Bucharest, Romania; 4 Institute of Macromolecular Chemistry “Petru Poni”, 41A Grigore Ghica Voda Alley, 700487 Iasi, Romania; E-Mail: shova@icmpp.ro; 5 Organic Chemistry Department, Faculty of Chemistry, University of Bucharest, 90-92 Panduri Street, 050663 Bucharest, Romania; E-Mail: c.paraschivescu@gmail.com; 6 Coordination Chemistry Department, Moldova State University, 60 Mateevici Street, 2009 Chisinau, Moldova; E-Mail: dociu1946@yahoo.com

**Keywords:** Schiff bases, copper(II) complex, crystal structure, EPR spectra, antimicrobial activity

## Abstract

A novel Schiff base, ethyl 4-[(E)-(2-hydroxy-4-methoxyphenyl)methylene-amino]benzoate (**HL**), was prepared and structurally characterized on the basis of elemental analyses, ^1^H NMR, ^13^C NMR, UV-Vis and IR spectral data. Six new copper(II) complexes, [Cu(L)(NO_3_)(H_2_O)_2_] (**1**), [Cu(L)_2_] (**2**), [Cu(L)(OAc)] (**3**), [Cu_2_ (L)_2_Cl_2_(H_2_O)_4_] (**4**), [Cu(L)(ClO_4_)(H_2_O)] (**5**) and [Cu_2_(L_2_S)(ClO_4_)(H_2_O)]ClO_4_·H_2_O (**6**) have been synthesized. The characterization of the newly formed compounds was done by IR, UV-Vis, EPR, FAB mass spectroscopy, elemental and thermal analysis, magnetic susceptibility measurements and molar electric conductivity. The crystal structures of Schiff base and the complex [Cu_2_(L_2_S)(ClO_4_)(H_2_O)]ClO_4_·H_2_O (**6**) have been determined by single crystal X-ray diffraction studies. Both copper atoms display a distorted octahedral coordination type [O_4_NS]. This coordination is ensured by three phenol oxygen, two of which being related to the µ-oxo-bridge, the nitrogen atoms of the azomethine group and the sulfur atoms that come from the polydentate ligand. The *in vitro* antimicrobial activity against *Escherichia coli* ATCC 25922, *Salmonella enteritidis*, *Staphylococcus aureus* ATCC 25923, *Enterococcus* and *Candida albicans* strains was studied and compared with that of free ligand. The complexes **1**, **2**, **5** showed a better antimicrobial activity than the Schiff base against the tested microorganisms.

## 1. Introduction

The synthesis of new compounds that are used for the treatment of infections with less secondary effects is a biomedical problem [1,2]. Recently research has focused increasingly on the synthesis of transition metal complexes with Schiff-type ligands, due to the biological properties that they present. Many compounds derived from Schiff bases exhibit antibacterial, antifungal, antitumor and anti-HIV activities [3,4,5,6,7,8,9,10,11,12,13,14]. Schiff bases derived from salicylaldehyde represent an important class of ligands due to their ability to be used in various fields [15,16,17,18,19]. Having a high capacity chelating and redox potential the positive Cu^2+^ ion is biologically active, and participates in many processes in the body [20,21,22]. Copper complexes are also among the most potent antiviral, antitumor and anti-inflammatory agents [23]. In addition, it was found that most of benzocaine-containing compounds are biologically active. Recent research has demonstrated that, these derivatives exhibit antimicrobial activity against different species [24,25,26]. Based on these observations and considering research in this area in the present study, we report the synthesis, characterization, antimicrobial studies of some copper(II) complexes containing a Schiff base ligand derived from 2-hydroxy-4-methoxybenzaldehyde and ethyl-4-aminobenzoate, with a special impetus on ligand structural investigations. The crystal structures of ligand and complex **6** were studied by X-ray diffraction. The biological properties of these compounds was tested for their antibacterial and antifungal activities against *Escherichia coli* ATCC 25922, *Salmonella enteritidis*, *Staphylococcus aureus* ATCC 25923 and *Candida albicans* strains.

## 2. Results and Discussion

### 2.1. Chemistry

The Schiff base **HL** was prepared by refluxing in ethanol an equimolar mixture of 4-aminobenzoic acid ethyl ester with 2-hydroxy-4-methoxybenzaldehyde. The structure of the formed Schiff bases was established by IR, ^1^H-NMR and ^13^C-NMR spectroscopies and by X-ray crystallography. All compounds were synthesized by direct reaction between ligand and the corresponding metal salts, while compound **6** was prepared by using a mixture of Cu(ClO_4_)_2_·6H_2_O and **HL** with addition of NaSCN. All complexes are microcrystalline solids. Also, the melting point values are greater than 220 °C. They are insoluble in organic solvents such as acetone and methanol, but soluble in DMF and DMSO. The molar conductivity values (8–12 ohm^−1^·cm^2^·moL^−1^) of the complexes **1**–**5** in 10^−3^ M solution DMF show that they are non-electrolytes and **6** (82 ohm^−1^·cm^2^·moL^−1^) is an electrolyte [27].

The thermal decompositions of the complexes **1**–**4** were studied by thermogravimetry (TG). The TG and TGD curves have three or four stages of mass loss. The first weight loss step correspond of two and four water molecules respectively, per one molecule of complex (7.26% for complex 1, 8.63% for complex 4). Thermal analyses data reveal that compounds **2** and **3** not are hydratated. The final residue was analyzed by IR spectroscopy, which confirms the formation of CuO (Figure S1).

**Figure 1:**
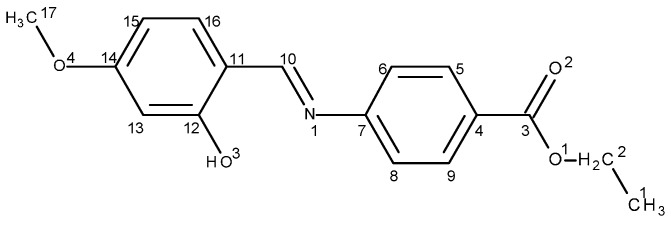
Schiff base ligand (**HL**).

**Figure 2:**
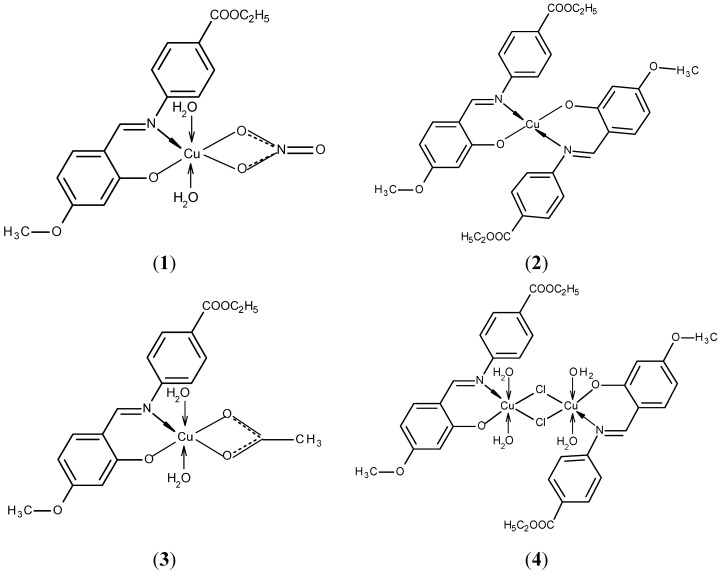

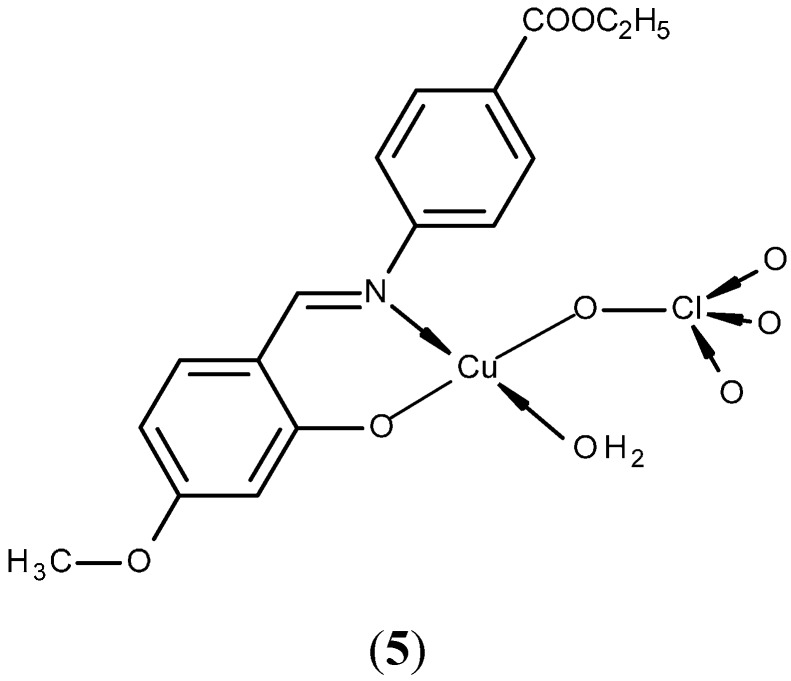
Proposed structures for the copper(II) complexes **1**–**5**.

The elemental analyses data of the Schiff base and complexes (reported in Section 3) are in agreement with structure of the ligand (Figure 1) and with structures of the complexes (Figure 2).

#### 2.1.1. Structural characterization of (**HL**)

**Figure 3:**
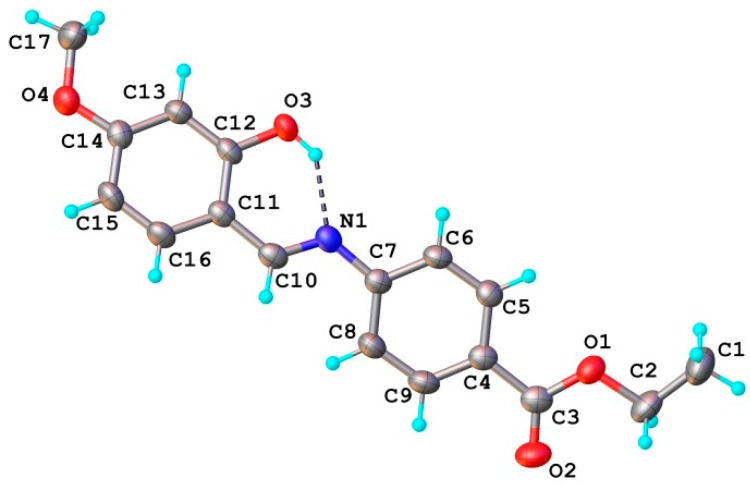
X-ray molecular structure of (**HL**). Thermal ellipsoids are drawn at 50% probability level. H-bond parameters: O3−H∙∙∙N1 [O3−H 0.86 Å, H∙∙∙N1 1.87 Å, O3∙∙∙N1 2.602(2) Å, O3−H∙∙∙N1 140.7°].

The ligand **HL** structure is shown in Figure 3. The planar configuration of **HL** molecule is stabilized by an intra-molecular O-H···N hydrogen bond which is observed in the majority of Schiff base ligands obtained from 2-hydroxybenzaldehyde [28].

**Figure 4:**
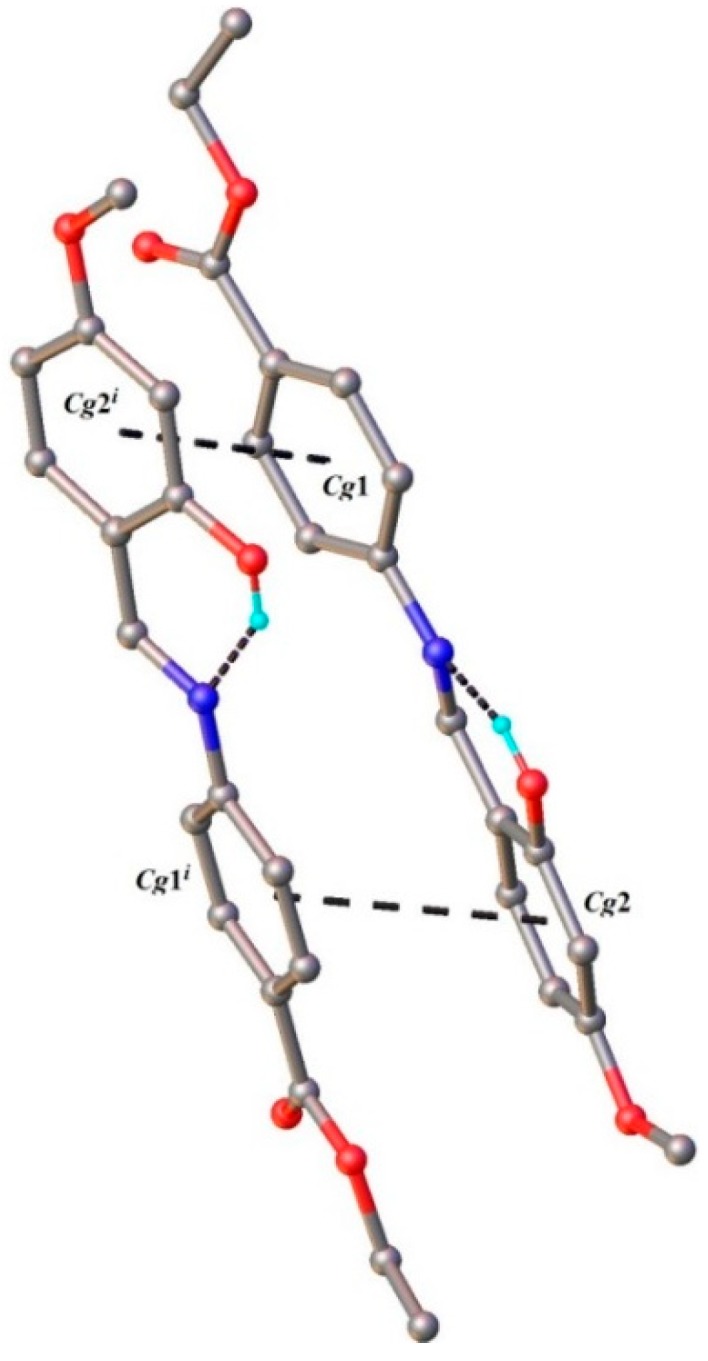
π-π staking interactions in the crystal structure (**HL**).

**Figure 5:**
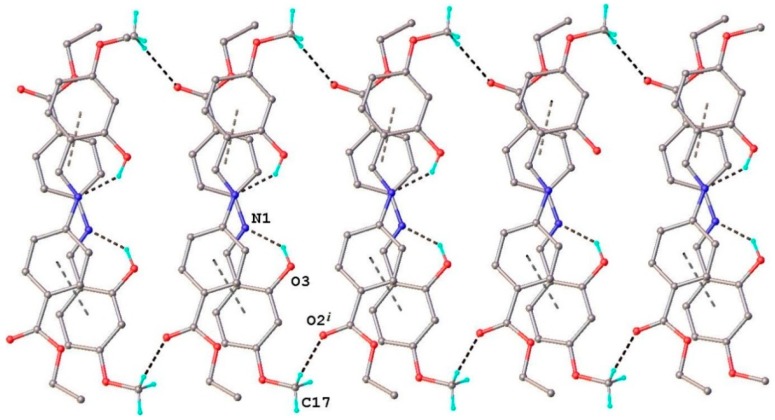
View of the supramolecular ribbon in the crystal structure (**HL)**. C17−H∙∙O2 [C17−H 0.96 Å, H∙∙∙O2 2.44 Å, C17∙∙∙O2(−*x*, −1 + *y*, 1.5 – *z*) 3.378(4) Å, C17−H∙∙∙O2 164.4°].

The crystal packing of **HL** is stabilized by π-π staking and C-H···O cooperative interactions. *Cg*1 (the centroid of the C-C9 ring) exhibits a stacking interaction with *Cg*2 (the centroid of the C11-C16 ring) of the adjacent molecule, related by two-fold axis, with a centroid-to-centroid distance of 3.851(4) Å. The formed dimeric units (Figure 4) are further associated due to the presence of weak C-H···O intermolecular interactions resulting into the formation of two-dimensional supramolecular ribbons, as the main crystal structure packing motif, depicted in Figure 5.

#### 2.1.2. Structural Characterization of [Cu_2_(L_2_S)_2_(ClO_4_)(H_2_O)]ClO_4_·H_2_O (**6**)

Selected bond lengths and angles for **HL** are presented in Table 1 and Table 2.

**Figure 6:**
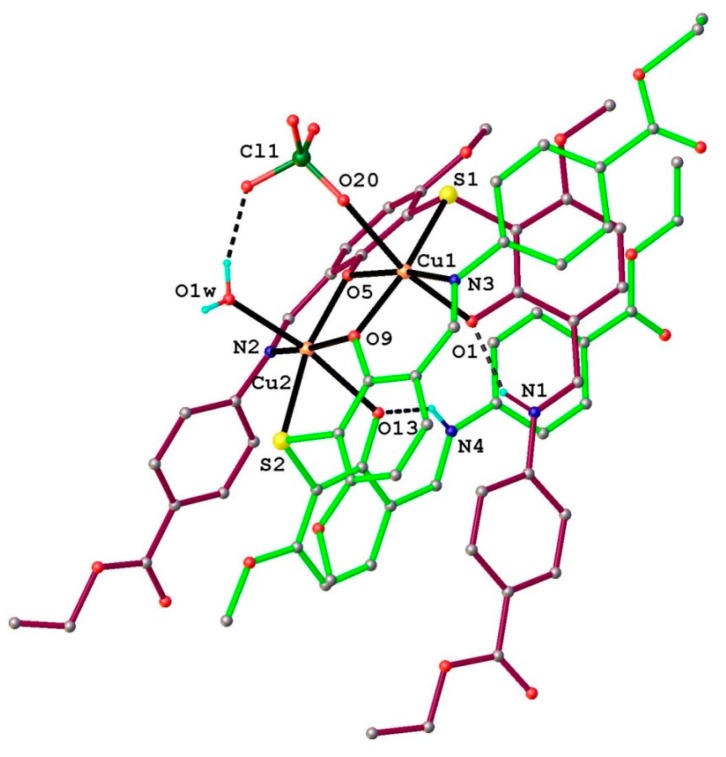
The X-ray structure of the complex cation [Cu_2_(L_2_S)_2_(ClO_4_)(H_2_O)]^+^ (**6**). H-bonds parameters: N1−H∙∙∙O1 [N1−H 0.86 Å, H∙∙∙O1 1.79 Å, N1∙∙∙O1 2.526(7) Å, N1−H∙∙∙O1 141.7°]. N4−H∙∙∙O13 [N1−H 0.85 Å, H∙∙∙O13 1.80 Å, N4∙∙∙O13 2.522(7) Å, N4−H∙∙∙O13 140.0°]. O1*w*−H∙∙∙O19 [N1−H 0.85 Å, H∙∙∙O19 1.93 Å, O1*w* ∙∙∙O19 2.74(1) Å, O1*w*−H∙∙∙O19 161.3°].

Complex **6** has a crystal structure formed from the complex binuclear cations [Cu_2_(L_2_S)_2_(ClO_4_)(H_2_O)]^+^, ClO_4_^−^ anions and H_2_O as solvate molecules 1:1:1 molar ratio. The structure of the complex cation is presented in Figure 6.

**Table 1:** Selected bond lengths (Å) and bond angles (°) for **HL**.

Bond Lengths (Å) for HL		Bond Angles (°) for HL	
O1-C2	1.445(2)	C3-O1-C2	116.8(2)
O1-C3	1.343(2)	O1-C2-C1	107.4(2)
O2C3	1.210(2)	O1-C3-C4	112.4(2)
O3-C12	1.349(2)	O2-C3-O1	122.7(2)
O4-C14	1.363(2)	O2-C3-C4	124.9(2)
O4-C17	1.425(2)	C6-C7-N1	116.7(2)
N1-C7	1.411(2)	C8-C7-N1	125.0(2)
N1-C10	1.284(2)	C10-N1-C7	122.0(2)
C12-C13	1.386(2)	N1-C10-C11	122.3(2)
C13-C14	1.382(2)	O3-C12-C11	121.1(2)
		O3-C12-C13	118.3(2)
		O4-C14-C13	124.7(2)
		O4-C14-C15	114.5(2)
		C14-O4-C17	117.6(2)

**Table 2:** Selected bond lengths (Å) and bond angles (°) for compound **6**.

**Bond Lengths (Å) for 6**			
Cu1-S1	2.406(2)	Cl1-O17	1.363(7)
Cu1-O9	1.952(4)	Cl1-O20	1.374(6)
Cu1-O5	1.941(4)	Cl1-O18	1.375(7)
Cu1-O1	2.244(4)	Cl1-O19	1.42(1)
Cu1-N3	1.957(5)	O5-C23	1.324(7)
Cu2-S2	2.404(2)	C6-O1	1.282(7)
Cu2-O9	1.947(4)	N2-C25	1.317(8)
Cu2-O5	1.965(4)	N2-C26	1.409(8)
Cu2-N2	1.956(5)	N3-C43	1.423(9)
Cu2-O13	2.323(4)	O13-C57	1.292(7)
Cu2-O1*w*	2.356(6)	S1-C7	1.769(6)
S2-C41	1.786(6)	S1-C24	1.788(6)
S2-C58	1.777(6)	O9-C40	1.320(7)
**Bond Angles (°) for 6**			
O9-Cu1-S1	161.5(1)	O5-Cu2-O1W	92.1(2)
O9-Cu1-O1	99.5(2)	N2-Cu2-S2	105.4(2)
O9-Cu1-N3	92.1(2)	N2-Cu2-O5	92.4(2)
O5-Cu1-S1	84.1(1)	N2-Cu2-O13	87.9(2)
O5-Cu1-O9	77.6(2)	N2-Cu2-O1W	95.9(2)
O5-Cu1-O1	91.9(2)	O13-Cu2-S2	76.8(1)
O5-Cu1-N3	169.7(2)	O13-Cu2-O1W	169.4(2)
O1-Cu1-S1	78.1(1)	O1W-Cu2-S2	92.6(2)
N3-Cu1-S1	106.1(2)	Cu2-O9-Cu1	101.9(2)
N3-Cu1-O1	88.8(2)	Cu1-O5-Cu2	101.7(2)
O9-Cu2-S2	84.6(1)	O17-Cl1-O20	108.8(5)
O9-Cu2-O5	77.2(2)	O17-Cl1-O18	113.2(6)
O9-Cu2-N2	168.8(2)	O17-Cl1-O19	104.4(8)
O9-Cu2-O13	89.4(2)	O20-Cl1-O18	114.7(4)
O9-Cu2-O1W	88.7(2)	O20-Cl1-O19	107.7(6)
O5-Cu2-S2	161.0(1)	O18-Cl1-O19	107.3(6)
O5-Cu2-O13	97.6(2)		

**Figure 7:**
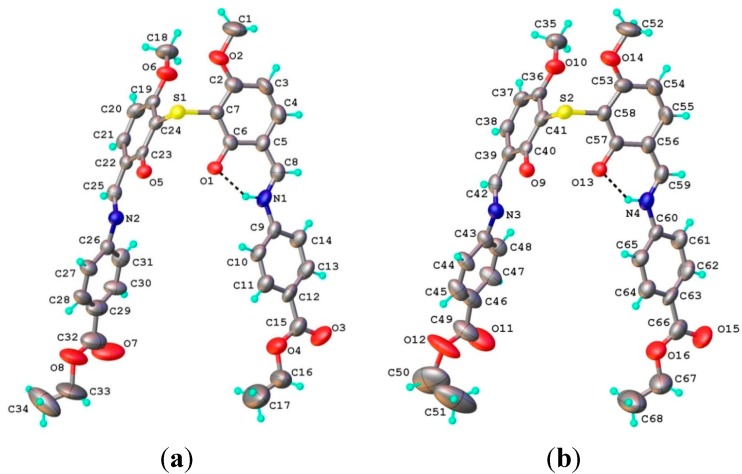
Structure of the two ligands **(**L_2_S) in the complex cation [Cu_2_(L_2_S)_2_(ClO_4_)(H_2_O)]^+^.

**Figure 8:**
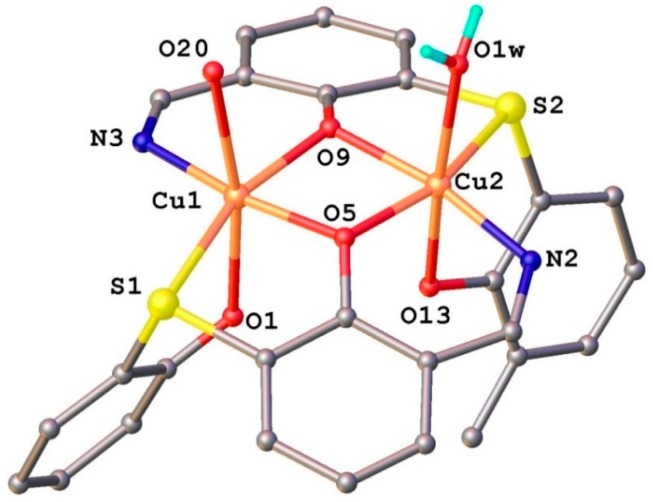
The coordination mode for the copper atoms of in the binuclear complex [Cu_2_(L_2_S)_2_(ClO_4_) (H_2_O)]^+^.

In the complex cation two Cu^2+^ ions, separated by 3.029(1) Å, are coordinated by two mono-deprotonated HL^−^ ligands, so that the charge balance is in agreement with the formation of species [Cu_2_(L_2_S)_2_ClO_4_H_2_O]^+^. The separate structure of the coordinated ligands along with the atomic labeling scheme is presented in Figure 7a,b. Both the ligands are identical from the chemical point of view and exhibit very close geometric parameters (Table 2). The localization of the hydrogen atoms using the difference density Fourier map has clearly shown that the non-coordinated azomethine atoms N1 and N4 are the sites of protonation associated with the hydrogen bonding towards phenolic oxygen atoms as acceptor (Figure 6 and Figure 7). The copper atoms adopt a slightly distorted octahedral [O_4_NS] coordination completed by ClO_4_^−^ anion and H_2_O molecule as monodentate ligands for Cu1 and Cu2, respectively (Figure 8).

**Figure 9:**
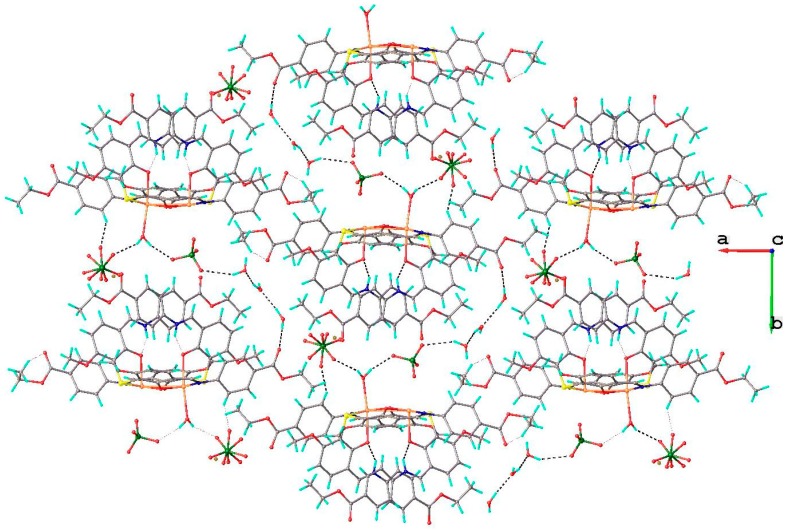
View of 2D supramolecular layer in the crystal structure **6**.

The crystal structure of complex **6** is characterized by the parallel packing of two dimensional supramolecular layers consolidated due to the numerous O-H···O and C-H···O hydrogen bonding, as shown in Figure 9.

#### 2.1.3. Infrared Spectra and Coordination Mode

The ligand and complexes have been characterized in detail by recording their IR spectra. The proposed assignments are based on previous results [28,29,30,31] and pertinent bibliography [32,33,34,35]. The ʋ(C=N) band of the ligand at 1622 cm^−1^ is found to be shifted to lower energies (1603–1595 cm^−1^) in the spectra of the complexes, indicating coordination via the azomethine nitrogen. The phenolic ʋ(C–O) stretching vibration in the free Schiff base is observed at 1113 cm^−1^, which is shifted by 10–21 cm^−1^ towards lower wave numbers in the complexes, thus indicating coordination of the phenolic oxygen to the Cu^2+^ ion [36].

In the IR spectra of complexes **1**, **4** and **5**, a considerable peak observed in the 3467–3420 cm^−1^ range supports the presence of ʋ(H_2_O) in the complexes [37]. The nitrate complex **1** has two bands at 1433 and 1182 cm^−1^ corresponding to ʋ_5_ and ʋ_1_, with a separation of 250 cm^−1^, and a medium band at 930 cm^−1^ assigned to ʋ_2_ of the nitrato group, values that indicate bidentate coordination [38,39].

The acetate complex **3** has two strong bands at 1530 and 1442 cm^−1^ corresponding to ʋ_as_(COO^−^) and ʋ_s_(COO^−^) with a difference between frequencies of 88 cm^−1^. This difference confirms the bidentate nature of the coordinated acetate [40].

The perchlorate complex **5** shows a band at 1108 cm^−1^ assignable to ʋ_3_(ClO_4_^−^) and a strong band at 1090 cm^−1^ assignable to ʋ_4_(ClO_4_^−^). The splitting of this band in two components indicates the presence of a monodentate perchlorate group [33,38].

#### 2.1.4. Electronic Spectra and Magnetic Studies

The tentative assignments of the significant electronic spectral bands of ligand and of complexes are presented in Table 3. In these spectra, there is an intense band at 33,330 cm^−1^ which is assigned to a π→π* transition originating in the phenyl ring [41]. The band at 28,570 cm^−1^ is attributed to an n→π* transitions originating in the -CH=N- chromophore [42]. In the spectra of the complexes these bands are shifted to lower energies (Figure S2).

**Table 3:** Electronic spectra (cm^−1^) and magnetic moments (BM) of the complexes **1**–**5**.

Metal Complex		Transitions d–d (cm^−1^)		µ_eff_ (BM)	Geometry
[Cu(L)(NO_3_)(H_2_O)_2_] (**1**)	^2^B_1g_→^2^A_1g_^2^ 10,500	^2^B_1g_→^2^B_2g_ 13,420	^2^B_1g_→^2^E_g_ 19,400	1.98	Octahedral distorted
[Cu(L)_2_] (**2**)	^2^B_2_→^2^E 11,100	^2^B_2_→^2^B_1_(^2^A_1_) 13,100	-	1.87	Pseudo-tetrahedral
[Cu(L)(OAc)] (**3**)	^2^B_2_→^2^E 10,980	^2^B_2_→^2^B_1_(^2^A_1_) 13,500	-	1.92	Pseudo-tetrahedral
[Cu_2_ (L)_2_Cl_2_(H_2_O)_4_] (**4**)	^2^B_1g_→^2^A_1g_ 11,000	^2^B_1g_→^2^B_2g_ 12,820	^2^B_1g_→^2^E_g_ 16 940	1.07	Octahedral distorted
[Cu(L)(ClO_4_)(H_2_O)] (**5**)	^2^B_2_→^2^E 9850	^2^B_2_→^2^B_1_(^2^A_1_) 12,200	-	1.90	Pseudo-tetrahedral

The electronic spectra for the Cu(II) complexes **2**, **3** and **5** exhibit two bands in the region 10,980–11,100 cm^−1^ respectively 13,150–13,880 cm^−1^, attributed to transitions d–d: ^2^B_2_→^2^E, ^2^B_2_→^2^B_1_(^2^A_1_), suggesting a distorted tetrahedral geometry [43,44]. The values of the magnetic momentum (1.87, 1.92 and 1.90 BM) indicate the existence of copper complexes under monomers form. The spectrum for complex **1** exhibit three bands at 10,500, 13,420 and 19,410 cm−1 corresponding to the transitions: ^2^B_1g_→^2^A_1g_^2^, ^2^B_1g_→^2^B_2g_, ^2^B_1g_→^2^E_g_, specific to the octahedral complexes with $d_{x^{2}-y^{2}}$ ground state [43].

The magnetic moment value 1.98 BM for complex **1** indicative of a non-bridging copper(II) complex. The electronic spectrum of complex **4** exhibit three bands at 11,000, 12,110 and 16,940 cm^−1^ corresponding to the transitions: ^2^B_1g_→^2^A_1g_, ^2^B_1g_→^2^B_2g_, ^2^B_1g_→^2^E_g_ suggesting a octahedral geometry [43].

The magnetic moment of the complex **4**, measured at room temperature, is 1.07 BM, per copper center. The subnormal magnetic moment indicates that the copper centers are anti-ferromagnetically coupled [45,46,47,48,49,50,51,52,53,54].

#### 2.1.5. Mass Spectra

The FAB mass spectra of Cu(II) complexes with base Schiff **HL** have been recorded (Table S1). The mass spectrum of ligand showing a peak at *m*/*z* = 300.12 corresponds to the molecular ion [M]^+^ (Figure S3).

[M]^+^ peak obtained from the complexes are as follow: *m*/*z* = 386.9 (**1**), *m*/*z* = 658.0 (**2**), *m*/*z* = 399.1 (**3**), *m*/*z* = 759.0 (**4**), *m*/*z* = 361.0 (**5**). The results of mass spectrometry are consistent with the proposed formulas for these compounds as the peaks observed in these spectra correspond to fragments resulting from the expected fragmentations of the compounds.

#### 2.1.6. EPR Spectra

**Figure 10:**
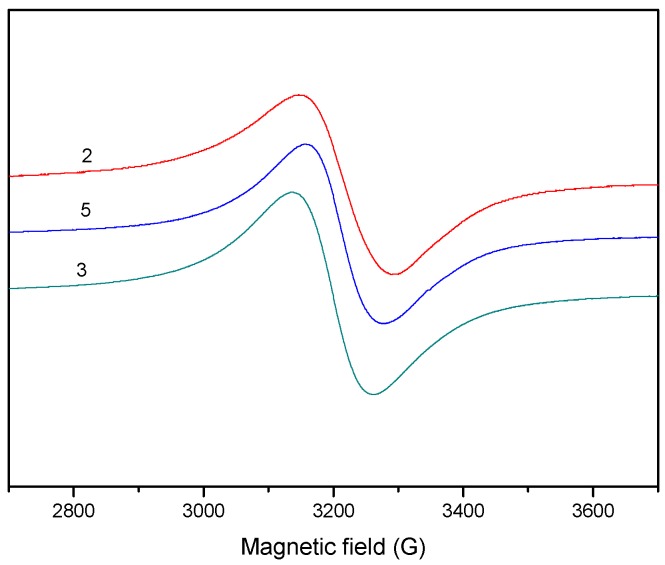
EPR spectra for the complexes **2**, **3** and **5** in powder, at room temperature.

The EPR spectra of the complexes in the polycrystalline state at 298 K and 77 K, were recorded at X-band, using 100 kHz field modulation. The g factors were quoted relative to the standard marker TCNE. The spectra of the compounds **1** and **4** in the polycrystalline state (293 K) show typical axial behavior with different *g*_ǁ_ and *g*_⊥_ values (Table 4). The geometric parameter G which is a measure of the exchange interaction between the copper centers in the polycrystalline compound is calculated using the equation: G = (*g*_ǁ_ − 2.0023)/(g_⊥_ − 2.0023), for axial spectra [55]. In the copper(II) complexes **1** and **4**, *g*_ǁ_ > *g*_⊥_ > 2.0023 and *G* values (2.77, 3.99) are consistent with a $d_{x^{2}-y^{2}}$ ground state [56]. The spectra of compounds **2**, **3** and **5** show only one broad signal at 2.097, 2.102 and 2.127, respectively, (Figure 10) consistent with tetrahedral geometry [57].

**Table 4:** EPR spectral parameters of the copper(II) complexes **1**–**5**.

	1	2	3	4	5
Polycrystalline (298K)					
*g*_ǁ_	2.230	-	-	2.132	-
g_⊥_	2.060	-	-	2.059	-
g_iso_	-	2.097	2.102	-	2.127
DMSO (77 K)					
*g*_ǁ_	2.400	2.290	2.289	2.239	2.282
g_⊥_	2.078	2.075	2.065	2.055	2.060
A_ǁ_	117.0	162.0	150.2	170.0	170.0
α^2^	0.802	0.800	0.779	0.772	0.817
β^2^	0.997	0.948	0.990	0.990	0.887
δ^2^	0.862	0.958	0.922	0.950	0.801
K_ǁ_	0.800	0.755	0.762	0.770	0.718
K_⊥_	0.692	0.760	0.715	0.735	0.658

**Figure 11:**
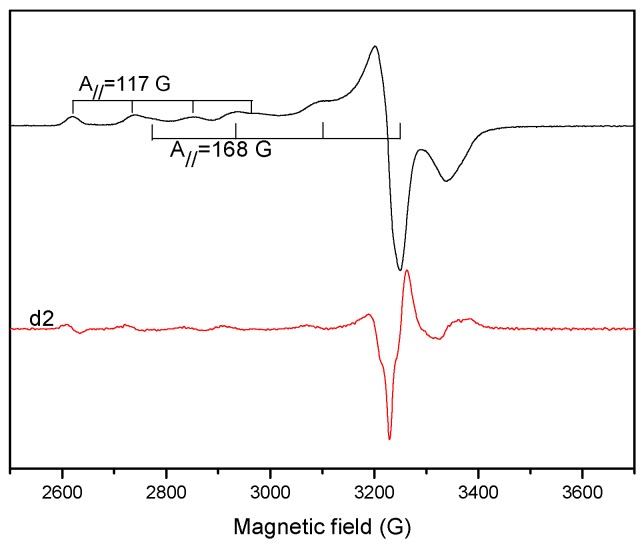
EPR spectrum of the complex **1**, in DMSO solution, registered at 77 K and (d2) second derivative spectra.

The solution of complexes were recorded in DMSO at 298 K and 77 K. They present well-resolved four hyperfine lines (Figure 11).

**Figure 12:**
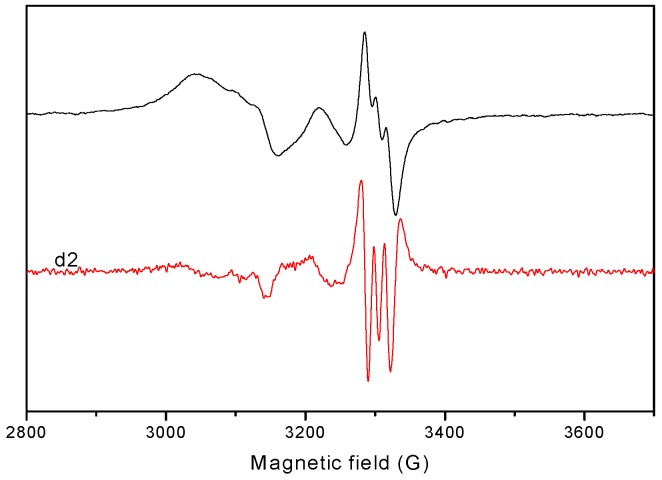
EPR spectrum (the derived signal) for complex **5** in DMSO solution at 77.

Spectra of the complexes **1**, **3** and **5** show three nitrogen superhyperfine lines in the perpendicular component (Figure 12).

**Figure 13:**
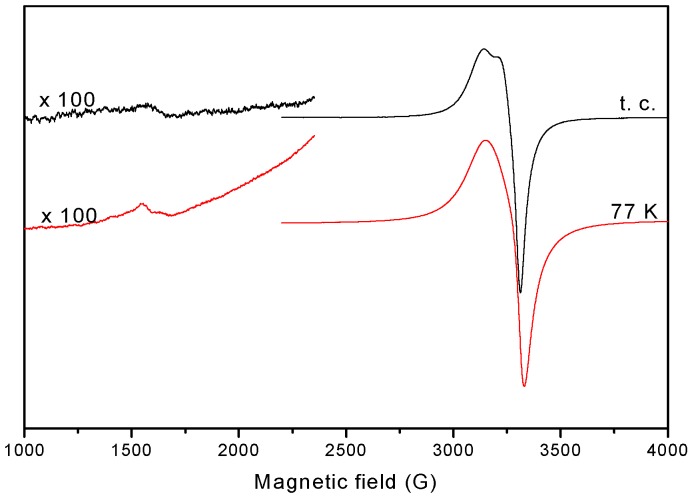
EPR spectrum of the complex **4**, in solution, at room temperature and 77 K.

The binuclear nature of **4** was confirmed by the presence of half-field signals at *ca.*1600 G (Figure 13).

EPR spectral assignments of the copper (II) complexes and orbital reduction parameters are shown in Table 4. The EPR parameters *g*_ǁ_, g_⊥_, A_ǁ_ (Cu) and the energies of the d–d transition were used to evaluate the bonding parameters α^2^, β^2^ and δ^2^, which may be regarded as measures of the covalency of the in-plane σ bonds, in-plane π bonds and out-of-plane π bonds [58,59]. The orbital reduction factors K_ǁ_ = α^2^β^2^ and K_⊥_ = α^2^δ^2^, were calculated using expressions reported elsewhere [60]. Hathaway has pointed out that for pure σ bonding, K_ǁ_ ~ K_⊥_ ~ 0.77 and for in-plane π -bonding, K_ǁ_ < K_⊥_, while for out-of-plane π-bonding, K_ǁ_ > K_⊥_ [61].

### 2.2. Biological Activity

The synthesized compounds and ligand were tested for their *in vitro* antibacterial and antifungal activity against *Escherichia coli* ATCC 25922, *Salmonella enteritidis*, *Staphylococcus aureus* ATCC 25923, *Enterococcus* and *Candida albicans* strains using the paper disc diffusion method [62] (for the qualitative determination) and the serial dilutions in liquid broth method [63] for determination of MIC values. Furaciline and nistatine were used as reference substances.

Data from Table 5 confirm the fact that both the ligand **HL** and the coordinative complexes of copper have a reduced bacteriostatic activity within the limits of concentrations 0.5–10.0 mg/mL toward the gram-positive and gram-negative bacteria. A more nuanced sensitivity toward the searched complexes has been proved by the gram-positive micro-organisms. The experimental data obtained demonstrate that the bacteriostatic activity of the coordinative complexes is influenced by the number of ligand molecules **HL** found out in the inner sphere and ligand nature. Therefore, growing of the number of ligand molecules and replacing the ions chloro- or acetate- with the perchlorate ion leads to a growing of the antimicrobial activity. The complexes [Cu(L)_2_] (**2**), [Cu(L)(ClO_4_)(H_2_O)] (**5**) and [Cu_2_(L_2_S)(ClO_4_)(H_2_O)]ClO_4_·H_2_O (**6**) develop bacteriostatic activity also toward *Candida albicans* in the area of concentrations 0.5–0.12 mg/mL, while the initial Schiff base **HL** which enters the inner sphere of these complexes doesn’t develop antifungal activity.

**Table 5:** Antibacterial activities of ligand **HL** and complexes **1**–**5** as MIC ^a^/MBC ^b^ values (mg/mL).

Compounds	*E. coli (G*−*)*		*S. enteritidis (G*−*)*		*S. aureus (G+)*		*Enterococcus (G+)*		*C. albicans*	
	MIC	MBC	MIC	MBC	MIC	MBC	MIC	MBC	MIC	MBC
C_17_H_17_NO_4_ (**HL^4^**)	> 10.0	>10.0	>10.0	>10.0	>10.0	>10.0	>10.0	>10.0	>10.0	>10.0
[Cu(L)(NO_3_)(H_2_O)_2_] (**1**)	>10.0	>10.0	>10.0	>10.0	0.5	>10.0	0.5	>10.0	0.5	>10.0
[Cu(L)_2_] (**2**)	>10.0	>10.0	>10.0	>10.0	0.5	>10.0	0.5	>10.0	0.12	>10.0
[Cu(L)(OAc)] (**3**)	>10.0	>10.0	>10.0	>10.0	>10.0	>10.0	0.5	>10.0	>10.0	>10.0
[Cu_2_ (L)_2_Cl_2_(H_2_O)_4_] (**4**)	>10.0	>10.0	>10.0	>10.0	0.5	>10.0	>10.0	>10.0	>10.0	>10.0
[Cu(L)(ClO_4_)(H_2_O)] (**5**)	0.5	>10.0	>10.0	>10.0	0.5	>10.0	0.5	>10.0	0.12	>10.0
Furacillinum	0.018	0.037	0.009	0.009	0.009	0.009	0.037	0.037	-	-
Nystatine	-	-	-	-	-	-	-	-	0.08	0.08

*E. coli* (Escherichia coli, ATCC 25922); *S. enteritidis* (Salmonella enteritidis); *S. aureus* (Staphylococcus aureus, ATCC 25923); *C. albicans* (Candida albicans); ^a^ MIC—Minimum inhibitory concentration; ^b^ MBC—Minimum bactericide concentration; G(−): Gram-negative bacteria; G(+): Gram-positive bacteria.

## 3. Experimental

### 3.1. General Information

All commercially available reagents and chemicals were of analytical- or reagent-grade purity and used as received. The chemical elemental analysis for the determination of C, H, N was done on a Carlo-Erba LA-118 microdosimeter (Lakewood, CA, USA). ^1^H-NMR and ^13^C-NMR spectra were recorded at room temperature on a Bruker DRX 400 spectrometer (Billerica, MA, USA) in DMSO-d_6_, using TMS as the internal standard. IR spectra were recorded on a Specord-M80 spectrophotometer (Leipzig, Germany) in the 4000–400 cm^−1^ region using KBr pellets. The complexes were studied by thermogravimetry (TG) in a static air atmosphere, with a sample heating rate of 10 °C/min using a STA 6000 Perkin Elmer (Waltham, MA, USA). Electronic spectra were recorded using the JascoV-670 spectrophotometer (Tokyo, Japan) in diffuse reflectance, using MgO dilution matrices. EPR spectra were recorded on polycrystalline powders and DMSO solutions at room temperature and 77 K with an MiniScope MS200, (Magnettech Ltd., Berlin, Germany), X-band spectrometer (9.3–9.6 GHz), connected to a PC equipped with a 100 KHz field modulation unit. High resolution mass spectra were recorded on a ThermoScientific (LTQ XLOrbitrap) spectrometer using APCI ionization technique and Orbital Ion Trap mass analyzer (Rockford, IL, USA).

The molar conductance of the complexes in *N,N'*-dimethylforamide solutions (10^−3^ M), at room temperature, were measured using a Consort type C-533 conductivity instrument. The magnetic susceptibility measurements were done at room temperature in the polycrystalline state on a Faraday magnetic balance (home made).

Crystallographic measurements were carried out on Oxford-Diffraction XCALIBUR E CCD diffractometer (Santa Clara, CA, USA) equipped with graphite-monochromated MoKα radiation. The single crystals were positioned at 40 mm from the detector and 197 and 273 frames were measured each for 20 and 30 s over 1° scan width for **HL** and **6**, respectively. The unit cell determination and data integration were carried out using the CrysAlis package of Oxford Diffraction [64]. Both structures were solved by direct methods using Olex2 [65] software with the SHELXS structure solution program and refined by full-matrix least-squares on *F*² with SHELXL-97 [66]. Atomic displacements for non-hydrogen atoms were refined using an anisotropic model. H atoms were placed at calculated positions and refined as riding atoms in the subsequent least-squares model refinements. The positional parameters of OH and NH hydrogen atoms were found from difference. The oxygen atoms in non-coordinated perchlorate anion were found to be severely disordered and their positional parameters were refined isotopically in combination with PART and SADI tools available in SHELXL Crystallographic data together with refinement details are summarized in Table 6.

**Table 6:** Crystallographic data, details of data collection and structure refinement parameters for **HL** and **6**.

Compound	HL	6
Empirical formula	C_17_H_17_NO_4_	C_68_H_66_Cl_2_Cu_2_N_4_O_26_S_2_
Formula weight	299.32	1617.35
Temperature/K	200	293
Crystal system	monoclinic	monoclinic
Space group	*C*2/*c*	*P*2_1_/*c*
*a*/Å	15.4089(7)	16.7582(11)
*b*/Å	6.4308(3)	26.2186(13)
*c*/Å	29.8710(16)	17.9269(12)
α/°	90.00	90.00
β/°	95.916(4)	107.710(8)
γ/°	90.00	90.00
*V*/Å^3^	2944.2(2)	7503.4(8)
*Z*	8	4
*D*_calc_/mg/mm^3^	1.351	1.432
μ/mm^−1^	0.097	0.774
Crystal size/mm^3^	0.2 × 0.1 × 0.1	0.35 × 0.35 × 0.1
θ_min,_ θ _max_ (°)	6.22 to 49.42	3.92 to 46.52
Reflections collected	4619	30514
Independent reflections	2404 [*R*_int_ = 0.0212]	10,753 [*R*_int_ = 0.0590]
Data/restraints/parameters	2404/0/201	10,753/29/942
GOF ^c^	1.010	1.028
*R*_1_ *^a^* (*I* > 2σ(*I*))	0.0434	0.0791
*wR*_2_^b^ (all data)	0.1020	0.2287
Largest diff. peak/hole/e Å^‒3^	0.11/−0.15	0.72/−0.87

*^a^* *R*_1_ = Σ||*F*_o_| − |*F*_c_||/Σ|*F*_o_|; *^b^* *wR*_2_ = {Σ[*w* (*F*_o_^2^ − *F*_c_^2^)^2^] /Σ[*w*(*F*_o_^2^)^2^ ]}^1/2^; *^c^* GOF = {Σ[*w*(*F*_o_^2^ − *F*_c_^2^)^2^] /(*n* – *p*)}^1/2^, where *n* is the number of reflections and *p* is the total number of parameters refined.

### 3.2. Chemistry

#### 3.2.1. Synthesis of the Schiff Base Ethyl 4-[(E)-(2-hydroxy-4-methoxyphenyl)methyleneamino] benzoate (**HL**)

A solution of 2-hydroxy-4-methoxybenzaldehyde (0.152 g, 1 mmoL) in ethanol (10 mL) was added to a solution of ethyl-4-aminobenzoate (0.165 g, 1 mmoL) in ethanol (20 mL). The mixture was stirred for 1 h at 25 °C, then refluxed for 4 h and kept at 4 °C for several days. The characteristic yellow precipitate obtained by Schiff base condensation was filtered out and kept for crystallization. Fine yellow crystals obtained upon slow evaporation at room temperature were characterized, including single crystal X-ray diffraction. Yield 81%. M.p. 125–127 °C. Anal. Calc. for C_17_H_17_NO_4_: C, 68.15; H, 5.67; N, 4.67. Found: C, 68.42; H, 5.43; N, 4.41%. The IR spectrum of the obtained ligand confirms the occurrence of the absorption band of 1622 cm^−1^ (st, intense), specific for the azomethinic group [39]. The ^1^H NMR spectra (CDCl_3_, d, ppm, J, Hz), recorded in CDCl_3_, for the ligand has the following signals confirming the structure of the ligand: ^1^H-NMR (CDCl_3_, δ, ppm, *J*, Hz): 1.40 (s, 3H, CH3); 3.84 (s, 3H, OCH3); 4.40 (s, 2H, CH_2_); 6.13 (d, 8.5, 1H, H-13); 6.50 (d, 8.5, 1H, H-15); 7.26 (s 1H, H-16); 7.60–8.15 (m, 4H, benzene); 8.53 (s, 1H, CH=N). ^13^C-NMR (CDCl_3_, δ, ppm): 14.48 (CH_3_); 55.63 (O-CH_3_); 61.13(OCH_2_);101.19 (C-13); 107.67 (C-15); 113.07 (C-11); 121.06 (C-8); 128.30 (C-4); 131.10 (C-5); 134.06 (C-9); 152.52 (C-N=CH);162.90 (C-OH); 164.20 (C=N); 164.63 (C-OCH_3_); 166.30 (C=O). The ligand is soluble in chloroform and dichloromethane.

#### 3.2.2. General Procedure for the Preparation of the Metal Complexes **1**–**6**

Complexes **1**–**5** were prepared by direct reaction between the ligand and the corresponding metal salts. Complex **6** was obtained by stirring a mixture of ligand, Cu(ClO_4_)_2_·6H_2_O and NaSCN.

*Synthesis of the complex [Cu(L)(NO_3_)(H_2_O)_2_]* (**1**): A methanol solution (15 mL) of the ligand HL (0.299 g, 1 mmoL) was added to Cu(NO_3_)_2_·3H_2_O (0.241g, 1 mmoL) dissolved in methanol (10 mL). The resulting solution was stirring for 6 h at 50 °C. The green colored solid was filtered, washed with methanol, diethylether and then dried *in vacuo*. Anal. Calc. for C_17_H_20_CuN_2_O_9_: C, 44.39; H, 4.35; N, 6.09; Cu, 13.81. Found: C, 44.60; H, 4.12; N, 5.88; Cu, 13.62%. M.wt.: 459.5; M.p. > 250 °C; Yield: 82%; Main IR peaks (KBr, cm^−1^): ʋ(OH) 3420; ʋ(C=O) 1705; ʋ (C=N) 1595; ʋ(Ar–OH) 1100; ʋ(Ar–O–C_aliphatic_) 1274, 1025; ʋ_5_ (NO_3_^−^) 1433; ʋ_1_(NO_3_^−^) 1182.

*Synthesis of the complex [Cu(L)_2_]* (**2**): Complex **2** was prepared similarly, using CuSO_4_·5H_2_O (1 mmoL). Brown solid. Anal. Calc. for C_34_H_32_CuN_2_O_8_: C, 61.86; H, 4.85; N, 4.24; Cu, 9.62. Found: C, 62.04; H, 4.52; N, 4.01; Cu, 9.38%. M.wt.: 659.5; M.p. > 250 °C; Yield: 87%; Main IR peaks (KBr, cm^−1^): ʋ(C=O) 1706; ʋ(C=N) 1603; ʋ(Ar–OH) 1092; ʋ(Ar–O–C_aliphatic_) 1272, 1023.

*Synthesis of the complex [Cu(L)(OAc)]* (**3**): Complex **3** was prepared similarly, using Cu_2_(OAc)_4_·(H_2_O)_2_ (1 mmoL). Brown solid. Anal. Calc. for C_19_H_19_CuNO_6_: C, 54.22; H, 4.51; N, 3.32; Cu, 15.10. Found: C, 54.63; H, 4.24; N, 3.08; Cu, 14.87%. M.wt.: 420.5; M.p. > 250 °C; Yield: 76%; Main IR peaks (KBr, cm^−1^): ʋ(C=O) 1703; ʋ(C=N) 1602; ʋ(Ar–OH) 1103; ʋ(Ar–O–C_aliphatic_) 1277, 1025; ʋ_as_(COO^−^)1530; ʋ_s_(COO^−^) 1442.

*Synthesis of the complex [Cu_2_ (L)_2_Cl_2_(H_2_O)_4_]* (**4**): Complex **4** was prepared similarly, using CuCl_2_·2H_2_O (1 mmoL). Light brown solid. Anal. Calc. for C_34_H_40_Cu_2_N_2_O_12_Cl_2_: C, 47.11; H, 4.61; N, 3.23; Cu, 14.66. Found: C, 46.83; H, 4.30; N, 3.02; Cu, 14.38%. M.wt.: 866; M.p. > 250 °C; Yield: 81%; Main IR peaks (KBr, cm^−1^): ʋ(OH) 3440; ʋ(C=O) 1702; ʋ(C=N) 1601; ʋ(Ar–OH) 1101; ʋ(Ar–O–C_aliphatic_) 1273, 1024.

*Synthesis of the complex [Cu(L)(ClO_4_)(H_2_O)]* (**5**): Complex **5** was prepared similarly, using Cu(ClO_4_)_2_·6H_2_O (1 mmoL). Brown solid. Anal. Calc. for C_17_H_18_CuNO_9_Cl: C, 42.58; H, 3.75; N, 2.92; Cu, 13.25. Found: C, 42.24; H, 3.52; N, 2.68; Cu, 13.03%. M.wt.: 479; M.p. > 250 °C; Yield: 79%; Main IR peaks (KBr, cm^−1^): ʋ(OH) 3467; ʋ(C=O) 1706; ʋ(C=N) 1599; ʋ(Ar–OH) 1093; ʋ(Ar–O–C_aliphatic_) 1276, 1021; ʋ_3_(ClO_4_^−^) 1108; ʋ_4_(ClO_4_^−^) 1090.

*Synthesis of the complex [Cu_2_(H_2_L_2_S)(ClO_4_)(H_2_O)]ClO_4_·H_2_O* (**6**): A solution of 2-hydroxy-4-methoxybenzaldehyde (0.152 g, 1 mmoL) in methanol (10 mL) was added to a solution of ethyl-4-aminobenzoate (0.165 g, 1 mmoL) in methanol (20 mL). The reaction mixture was refluxed for 3 h. Copper(II) perchlorate hexahydrate (0.340 g, 1 mmoL) dissolved in methanol was added and the mixture was refluxed for 1 h. Finally 5 mL methanolic solution of NaSCN (0.162 g, 2 mmoL) was added dropwise, the mixture was refluxed for another 1 h and the resulting solution was then filtered. Reddish brown single crystals suitable for structure determination were obtained from methanolic solution within few days. Anal. Calc. for C_68_H_66_Cl_2_Cu_2_N_4_O_26_S_2_: C, 50.46; H, 4.08; N, 3.46; Cu, 7.91. Found: C, 50.86; H, 3.82; N, 3.24; Cu, 7.82%. M.wt.: 1617; M.p. > 250 °C; Yield: 83%; Main IR peaks (KBr, cm^−1^): ʋ(OH) 3450; ʋ(C=O) 1702; ʋ(C=N) 1596; ʋ(Ar–OH) 1095; ʋ(Ar–O–C_aliphatic_) 1272, 1025; ʋ_3_(ClO_4_^−^) 1106; ʋ_4_(ClO_4_^−^) 1090.

### 3.3. Antibacterial Activity

The antibacterial activity of complexes and also of their prototype furaciline has been determined under liquid nutritive environment [2% of peptone bullion (pH 7.0)] using successive dilutions method [67,68,69]. *Escherichia coli*, *Salmonella enteritidis*, *Staphylococcus aureus*, *Enterococcus* stems were used as reference culture for *in vitro* experiment. The dissolution of studied substances in dimethylformamide, microorganisms cultivation, suspension obtaining, determination of minimal inhibition concentration (MIC) and minimal bactericide concentration (MBC) have been carried out according to the method previously reported.

### 3.4. Antifungal Bioassay

Antimycotic properties of the complexes were investigated *in vitro* on laboratory stem *Candida albicans*. The activity has been determined in liquid Sabouroud nutritive environment (pH 6.8). The inoculates were prepared from fungal stems which were harvested from 3–7 day-old cultures. Their concentration in suspension was (2–4) × 10^6^ colony forming units per milliliter. Sowings for fungi and micelles were incubated at 37 °C during 7 and 14 days, respectively.

## 4. Conclusions

The analytical and physico-chemical analyses confirm the composition and the structure of the newly obtained complex combinations. In all the complexes, the ligand base Schiff, **HL** acts as mono-negative bidentate around the metal ion. The structure of the ligand **HL** and complex **6** has been determined by single-crystal X-ray diffraction. This fact indicates for complex **6** a bi- nuclear system where every atom of Cu(II) is hexacoordinated through the medium of 4 atoms of oxygen, one atom of nitrogen and one atom of sulfur. The two atoms of copper (II) are united each other through the medium of two atoms of oxygen coming from the hydroxyl groups grafted on the aldehyde nucleus.

The EPR parameters *g*_ǁ_, g_⊥_, A_ǁ_ and the energies of d–d transitions were used to evaluate the bonding parameters. The orbital reduction factors indicate the presence of out-of-plane π-bonding for complexes **1**, **3**–**5** and of some in-plane π-bonding for the complex **2**.

The antimicrobial activity data obtained indicate that the activity of the metal complexes is higher than ligands. The structure of the compounds tested has an effect on antimicrobial activity.

## Supplementary Materials

Supplementary materials can be accessed at: http://www.mdpi.com/1420-3049/20/04/5771/s1.

## Appendix

CCDC 1005531 and 1005532 contains the supplementary crystallographic data for C_17_H_17_NO_4_ (**HL**) and C_68_H_66_Cl_2_Cu_2_N_4_O_26_S_2_ (**6**). These data can be obtained free of charge via http://www.ccdc.cam.ac.uk/data_request/cif or from the Cambridge Crystallographic Data Centre, 12 Union Road, Cambridge CB2 1EZ, UK; Fax: +44-1223-336-033; or E-Mail: deposit@ccdc.cam.ac.uk.
