# Supplementary material for: Synthesis, Characterization, Crystal Structure and Antimicrobial Activity of Copper(II) Complexes with the Schiff Base Derived from 2-Hydroxy-4-Methoxybenzaldehyde
Source: Molecules. 2015 Apr 2;20(4):5771–92. doi: 10.3390/molecules20045771 (PMC6272500; doi:10.3390/molecules20045771)
Supplement: Supplementary file 1 [file molecules-20-05771-s001.pdf]

Supplementary Materials

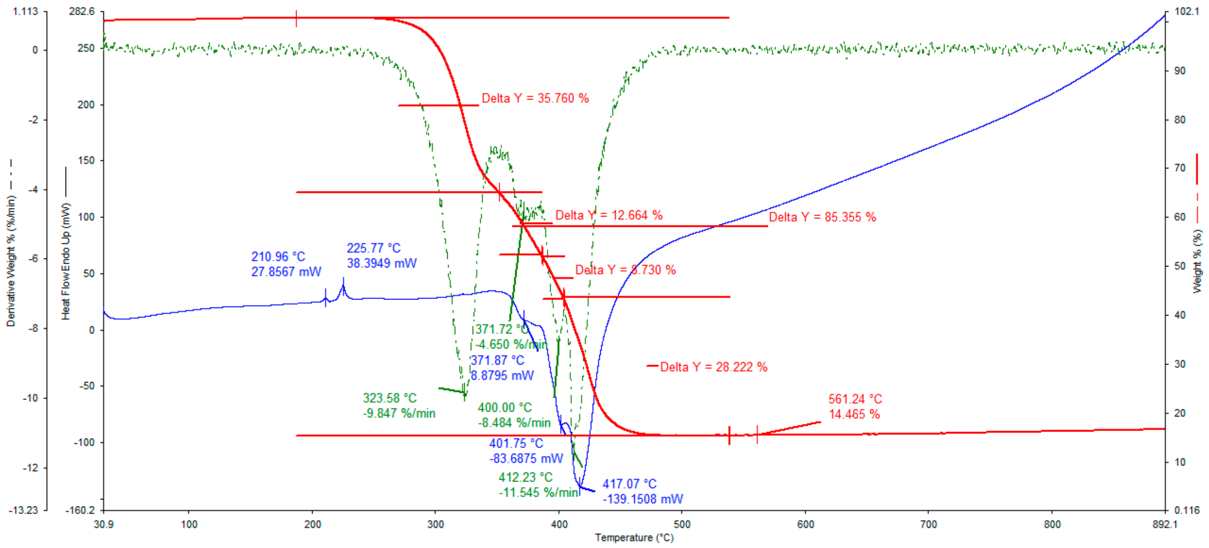

(**a**)

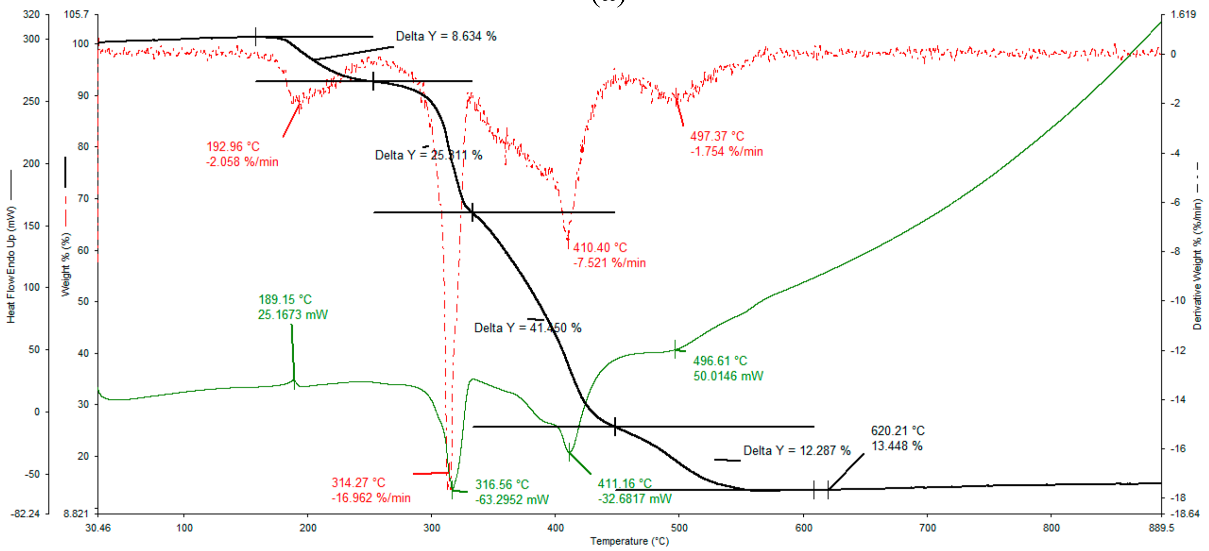

(**b**)

Figure S1. Thermogravimetric analysis complexes of (**a**) **2**, and (**b**) **4**.

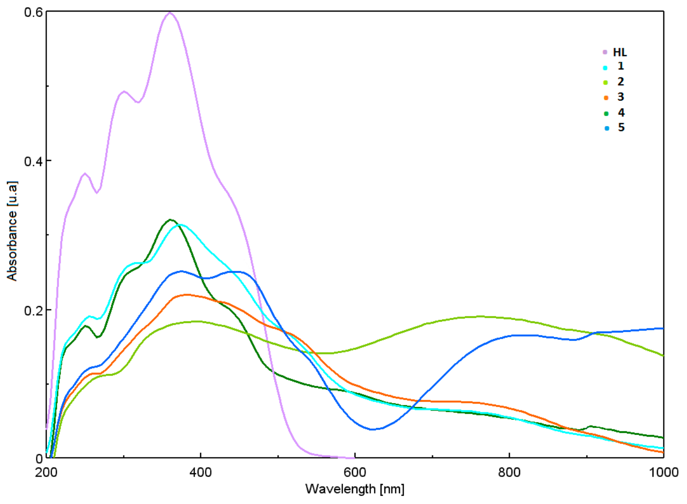

Figure S2. Electronic spectra of complexes **1**–**5**.

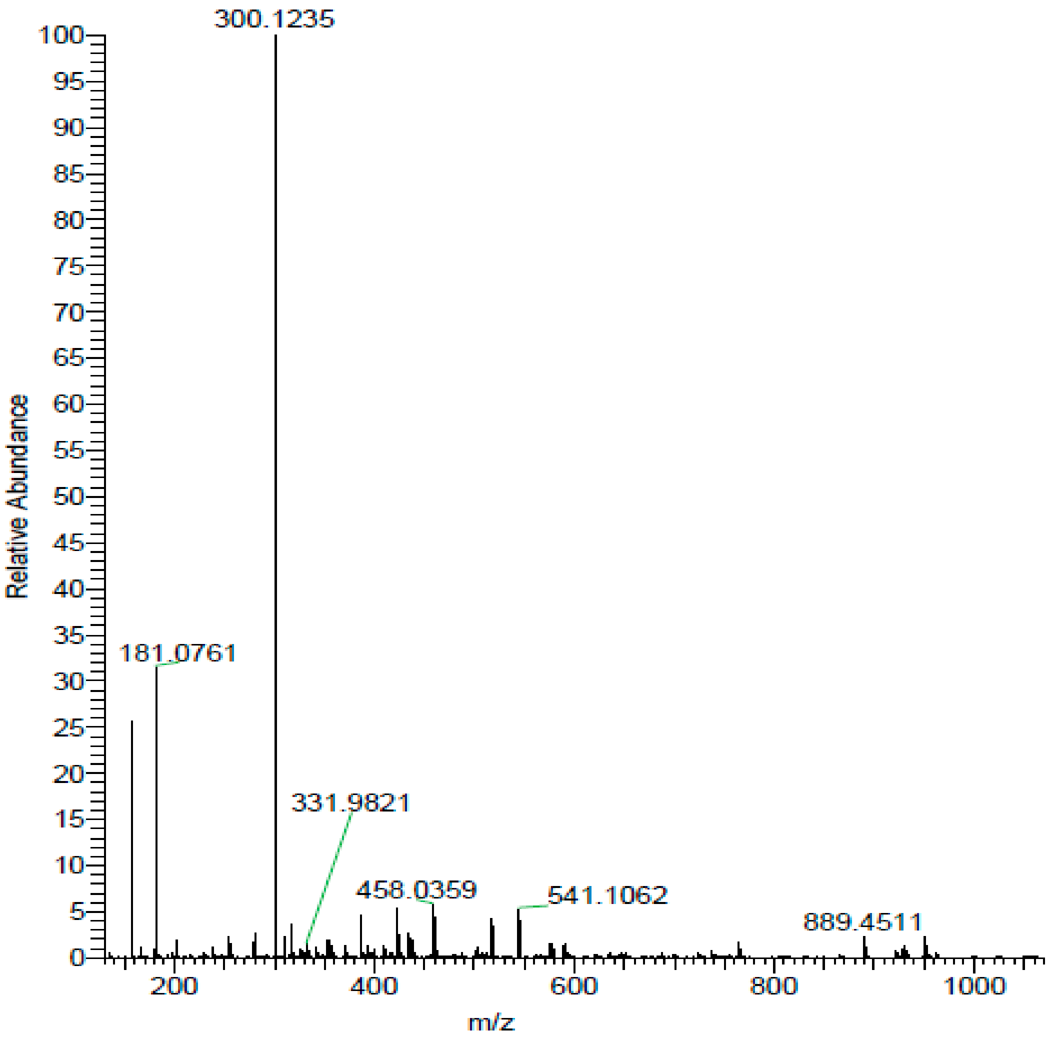

Figure S3. Mass spectra of Schiff base (**HL**).

Table S1. FAB mass spectral data of complexes **1**–**5**.

Molecular Formula	Mw (g/moL)	Molecular Ion Peak $[M]^+$	The Peaks due to Complex Fragmentation			
$[Cu(L)(NO_3)(H_2O)_2]$ (**1**)	459.5	386.9	253.9	308.9	331.9	343.1
$[Cu(L)_2]$ (**2**)	659.5	658.0	308.9	355.9	506.5	576.0
[Cu(L)(OAc)] (**3**)	420.5	399.1	300.1	334.2	366.2	383.2
$[Cu_2 (L)_2Cl_2(H_2O)_4]$ (**4**)	866	759.0	300.1	331.9	461.6	544.8
$[Cu(L)(ClO_4)(H_2O)]$ (**5**)	479	361.0	257.9	286.1	317.9	343.1
